# CHAC1 inactivation is effective to preserve muscle glutathione but is insufficient to protect against muscle wasting in cachexia

**DOI:** 10.1371/journal.pone.0283806

**Published:** 2023-04-04

**Authors:** Junjie Li, Mingjian Lu, Youngwook Ahn, Kevin Cao, Cynthia A. Pinkus, John C. Stansfield, Zhidan Wu, Bei B. Zhang

**Affiliations:** 1 Internal Medicine Research Unit, World Research, Development and Medical, Pfizer, Inc., Cambridge, Massachusetts, United States of America; 2 Emerging Science & Innovation, World Research, Development and Medical, Pfizer, Inc., Cambridge, Massachusetts, United States of America; 3 Early Clinical Development, World Research, Development and Medical, Pfizer, Inc., Cambridge, Massachusetts, United States of America; University of North Carolina at Greensboro, UNITED STATES

## Abstract

Muscle wasting is one of the main characteristics of cachexia associated with cancer and other chronic diseases and is often exacerbated by antineoplastic agents. Increased oxidative stress is associated with muscle wasting, along with depletion of glutathione, the most abundant endogenous antioxidant. Therefore, boosting endogenous glutathione has been proposed as a therapeutic strategy to prevent muscle wasting. Here, we tested this hypothesis by inactivating CHAC1, an intracellular glutathione degradation enzyme. We found CHAC1 expression is increased under multiple muscle wasting conditions in animal models, including fasting, cancer cachexia, and chemotherapy. The elevation of muscle Chac1 expression is associated with reduced glutathione level. CHAC1 inhibition via CRSPR/Cas9 mediated knock-in of an enzyme inactivating mutation demonstrates a novel strategy to preserve muscle glutathione levels under wasting conditions but fails to prevent muscle wasting in mice. These results suggest that preserving intracellular glutathione level alone may not be sufficient to prevent cancer or chemotherapy induced muscle wasting.

## Introduction

Cachexia is a devastating condition in patients with advanced cancer and other chronic illnesses. It is characterized by unintentional weight loss predominantly from loss of skeletal muscle and adipose tissue [1–3]. While appetite stimulant has been shown to reverse fat loss to some extent [4], reversal of muscle loss and functional impairment remains a challenge. Muscle quality and function are important determinants of quality of life and predictors of mortality in patients. Preserving muscle mass and function remains a major unmet medical need for patients with cancer cachexia. Potential causes of muscle wasting are multifactorial, including abnormal energy metabolism, excess protein breakdown through ubiquitin-mediated proteolysis and autophagy, inflammation, and oxidative stress [5, 6]. Increased oxidative stress has been known for decades to be associated with muscle wasting in cancer cachexia preclinically and in cancer patients [7, 8]. Oxidative stress exacerbates muscle wasting through multiple mechanisms, including decreasing protein synthesis by downregulating PI3K/Akt/mTOR pathway [9], increasing protein degradation by activation of proteasome and autophagy [10], impairing mitochondrial function [11], and degeneration of the neuromuscular junction [12]. Targeting excessive oxidative stress has been attempted to prevent muscle wasting under cachectic conditions, although evidence based efficacy in preclinical and clinical setting is yet to be established.

Oxidative stress is a condition of disturbed redox homeostasis either by excessive production of reactive oxygen species (ROS) or reactive nitrogen species (RNS), or by suppressed antioxidant mechanisms. Preclinically, increased level of protein oxidation and lipid peroxidation products (e.g., malondialdehyde (MDA) and 4-hydroxy-2-nonenal (HNE)) have been reported in skeletal muscles of rats bearing the Yoshida AH-130 hepatoma [13] or Walker 256 tumor [14] and mice bearing C26 tumors [11]. On the other hand, expression of antioxidant enzymes, such as superoxide dismutase (SOD), glutathione peroxidase (GPX) and catalase, were found to be downregulated in skeletal muscles of mice bearing cachectic tumors [15]. Clinically, increased oxidative stress as evidenced by protein carbonylation and lipid peroxidation was reported in cachectic lung cancer patients as well as Chronic Obstructive Pulmonary Disease (COPD) patients [16].

Given that increased oxidative stress is a common feature of skeletal muscle in cachexia, combating oxidative stress by supplementation of nutritional antioxidants has been explored as a therapeutic strategy for cancer cachexia preclinically and clinically. Antioxidants, including α-tocopherol [14, 17], cysteine [18, 19], N-acetylcysteine [20], pyrroloquinoline quinone [21], and ornithine [18], have been shown to be effective in suppressing oxidative stress in skeletal muscles in various preclinical cancer cachexia models. However, no clear demonstration of improvement in skeletal muscle mass or function was shown in these studies. Cysteine-rich protein showed clinical benefits in improving weight loss in lung cancer patients, though no oxidative stress related parameters were measured [22]. Despite these advances, nutritional supplementation of antioxidants remains as a controversial strategy for cancer cachexia treatment due to incomplete understanding of mechanisms of action and potential side effects. Strategies to boost the endogenous antioxidant defenses are highly desired.

Glutathione is the most abundant low-molecular-weight antioxidant in most cells. As substrate of GPX, reduced form of glutathione (GSH) detoxifies ROS/RNS and free radicals [23]. Glutathione level was found to be decreased in skeletal muscles of mice bearing MCA-105 fibrosarcoma [19] or C26 tumors [11]. Under stress or pathological conditions, cellular glutathione depletion can occur by efflux and degradation through γ-glutamyl transpeptidase (GGT) at the plasma membrane [24] or by intracellular breakdown. Recently, ChaC glutathione specific gamma-glutamylcyclotransferases (CHACs), including CHAC1 and CHAC2, were identified as intracellular glutathione degradation enzymes [25–27], contributing to the depletion of intracellular glutathione under stressed conditions. Compared to CHAC2, the catalytic efficiency of CHAC1 is 10–20 fold higher [27]. While human CHAC2 expression is ubiquitous, CHAC1 expression is highly enriched in skeletal muscle. Furthermore, human CHAC1, but not CHAC2, is specifically induced by ER stress [25, 26].

In this study, we tested the hypothesis that CHAC1 inhibition may preserve the intracellular glutathione in skeletal muscles and thus represent a novel strategy to treat muscle wasting in cachexia conditions. Importantly, we found that CHAC1 expression is significantly upregulated in skeletal muscles under various wasting conditions, including cancer induced cachexia, chemo-agent cisplatin induced cachexia, and fasting induced muscle atrophy. To test the hypothesis, we generated a CHAC1 knock-in (KI) mouse line with a point mutation introduced into the Chac1 genomic locus that inactivates its glutathione degradation activity. CHAC1 inactivation resulted in increased muscle glutathione levels under basal and wasting conditions. However, it did not protect the mice from muscle wasting in Pan02 cancer induced cachexia or cisplatin induced cachexia models. Our studies reveal a novel strategy to preserve muscle glutathione but also suggest that preserving glutathione alone may not be sufficient to prevent cancer or chemotherapy induced muscle wasting.

## Results

### Increased Chac1 expression and glutathione depletion in skeletal muscle under wasting conditions

**Fig 1:**
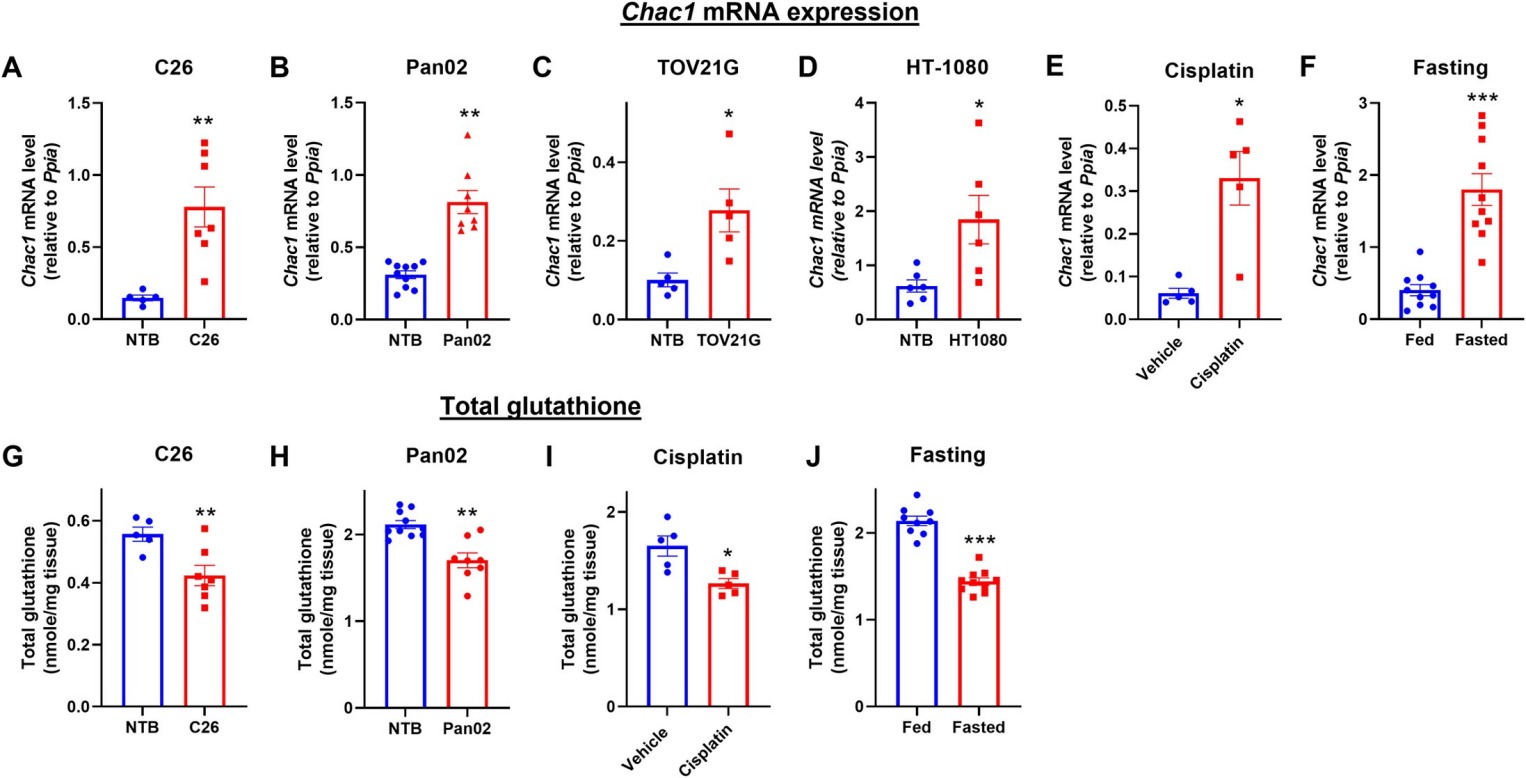
*Chac1* mRNA is upregulated under muscle wasting along with reduced glutathione level. **A-F.** *Chac1* mRNA expression measured in gastrocnemius muscles of **A.** C26 colon cancer cachexia model in Balb/c mice, **B.** Pan02 pancreatic cancer cachexia model in C57Bl/6 mice, **C.** TOV21G ovarian cancer cachexia model in NOD SCID mice, **D.** HT-1080 fibrosarcoma cachexia model in NOD SCID mice, **E.** chemotherapy cisplatin induced cachexia model in C57Bl/6 mice, and **F.** fasting induced muscle atrophy model in C57Bl/6 mice. **G-J.** Total glutathione measured in gastrocnemius muscles of **G.** C26 colon cancer cachexia model, **H.** Pan02 pancreatic cancer cachexia model, **I.** chemotherapy cisplatin induced cachexia model, and **J.** fasting induced muscle atrophy model. Results are shown as mean ± SEM with individual data plotted. Statistical significances were indicated as: ^*^ *P* < 0.05, ^**^ *P* < 0.01, ^***^ *P* < 0.001.

To explore the role of CHAC1 in muscle wasting, we examined the *Chac1* mRNA level in skeletal muscles in various wasting conditions. As shown in **Fig 1**, *Chac1* mRNA levels were upregulated 2-5-fold in skeletal muscles of tumor-bearing mice in four cancer cachexia models characterized previously [28–31], including colon cancer C26 (**Fig 1A**), pancreatic cancer Pan02 (**Fig 1B**), ovarian cancer TOV21G (**Fig 1C**), and fibrosarcoma HT-1080 (**Fig 1D**), when compared to nontumor bearing (NTB) mice. Additionally, *Chac1* mRNA increased in skeletal muscles of chemotherapeutic agent, cisplatin, treated mice (**Fig 1E**), as well as in skeletal muscles of mice after overnight fasting compared to non-fasted mice (**Fig 1F**). Correspondingly, we measured total glutathione levels in the same skeletal muscle tissues. Total glutathione levels were reduced 10–30% in skeletal muscles of C26 tumor-bearing mice (**Fig 1G**), Pan02 tumor-bearing mice (**Fig 1H**), cisplatin treated mice (**Fig 1I**), and fasted mice (**Fig 1J**) when compared to their respective control mice. The inverse association between changes in Chac1 mRNA expression level and glutathione level suggests a role of CHAC1 in glutathione depletion in skeletal muscle.

### Generation and characterization of enzyme inactive *Chac1*^E116A^ knock-in mice

To test the role of CHAC1 in vivo, we generated Chac1 knock-in mice that have lost enzymatic activity of Chac1 but with its protein expression retained. Glutamate at the 115^th^ position of human CHAC1 is critical to its glutathione degradation activity [26]. Overexpression of WT hCHAC1 depleted ~90% of intracellular glutathione in HEK-293 cells, while overexpression of either E115A or E115Q mutant had no effect on glutathione level (**S1A Fig**), consistent with previous reports that these mutations disrupt CHAC1 enzyme activity. Since the E115A mutant was expressed at levels comparable to the WT CHAC1, (**S1B Fig**), the E116A mutation (equivalent to E115A in human CHAC1) was used to generate the Chac1 enzyme inactive knock-in mice through CRSPR/Cas9-mediated genome editing. Genome sequencing of N1 heterozygotes confirmed successful introduction of the E116A mutation (GAA>GCC) (**S2 Fig**).

**Fig 2:**
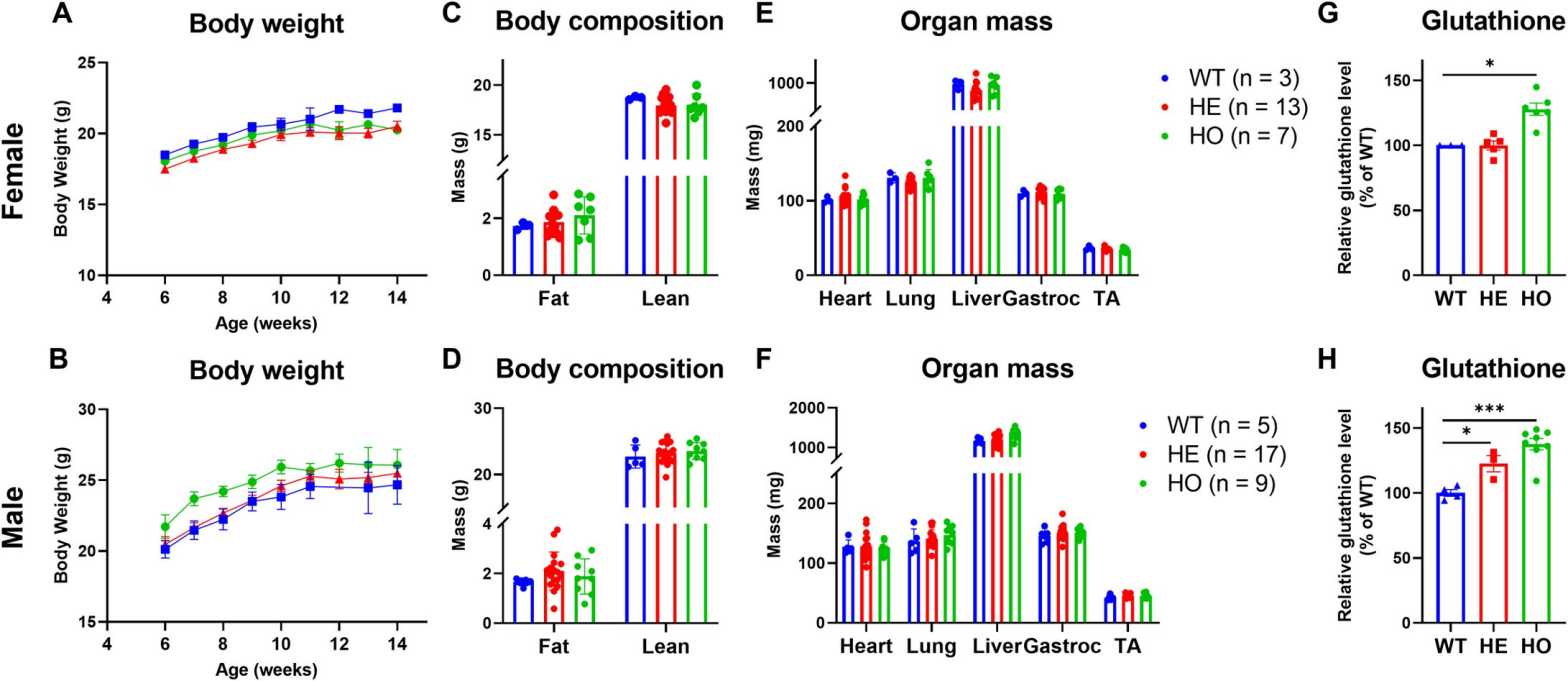
Normal development of the *Chac1*^E116A^ KI mice. **A, B.** Body weight curves from 6 weeks old to 14 weeks old, **C, D.** Body compositions in fat and lean mass, and **E, F.** Heart, lung, liver, gastrocnemius, and tibialis anterior (TA) muscle mass in WT, heterozygous (HE) and homozygous (HO) *Chac1*^E116A^ knock-in mice. **G, H.** Total glutathione measured in gastrocnemius muscles of WT, HE and HO KI mice. Data in top panels (A, C, E, G) were from females and the data in bottom panels (B, D, F, H) were from males. Results are shown as mean ± SEM with individual data plotted. Statistical significances were indicated as: ^*^ *P* < 0.05, ^***^ *P* < 0.001.

Baseline characterization of the WT, heterozygous (HE) and homozygous (HO) Chac1 KI mice showed no obvious abnormality in growth (**Fig 2A and 2B**), body composition (**Fig 2C and 2D**), and weight of heart, lung, liver, and skeletal muscles (gastrocnemius and tibialis anterior (TA)) (**Fig 2E and 2F**) in both females and males. The KI mice were fertile in both sexes. Moreover, we did not observe any obvious abnormality in a second E116A KI line and a knock-out line (1 bp insertion leading to premature truncated protein) (data not shown), suggesting Chac1 is not an essential gene for development in mice. Consistent with inhibition of CHAC1 enzyme activity, glutathione levels in gastrocnemius muscle of the KI mice were slightly increased in heterozygotes and significantly increased in homozygotes (28% in female and 38% in male) (**Fig 2G and 2H**). In contrast, no difference in glutathione levels was observed in heart or liver between homozygous and WT mice (**S3A and S3B Fig**), consistent with low expression of Chac1 in these tissues. These data indicate CHAC1 is a key regulator of glutathione levels in skeletal muscles, but may not in other organs, likely due to differential tissue expression of Chac1. These data also demonstrate the effectiveness of inactivating CHAC1 as a novel strategy to preserve muscle glutathione.

### CHAC1 inactivation preserves glutathione in skeletal muscle under fasting condition

**Fig 3:**
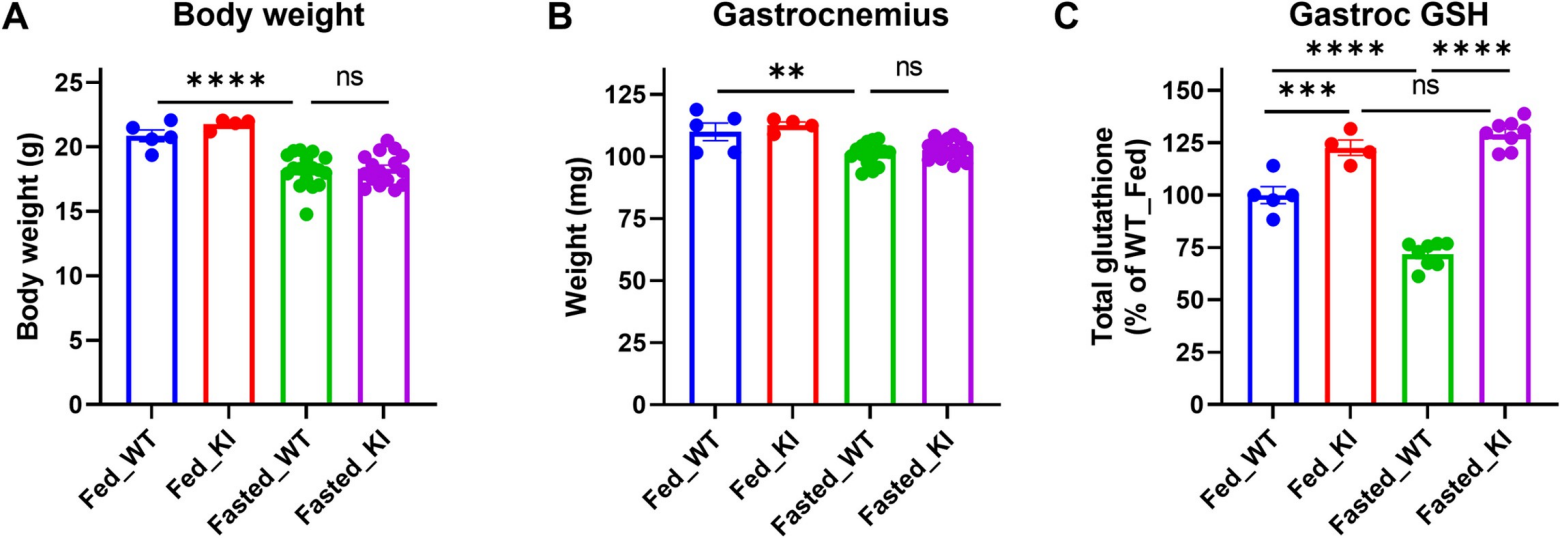
Chac1 inactivation preserves muscle glutathione but does not protect against fasting induced muscle atrophy. **A.** Body weight, and **B.** Gastrocnemius muscle weight measured after 24-h of fasting. **C.** Total glutathione measured in gastrocnemius muscle tissues. Results are shown as mean ± SEM with individual data plotted. Statistical significances were indicated as: ^ns^ not significant, ^*^ *P* < 0.05, ^**^ *P* < 0.01, ^***^ *P* < 0.001.

Considering a drastic upregulation of *Chac1* mRNA expression and reduction of glutathione level in skeletal muscle under fasting condition (**Fig 1F**), we explored the potential physiological role of CHAC1 in response to fasting. To achieve a biologically significant muscle atrophy phenotype, the mice were fasted for 24 h [32]. Body weight was 13–16% lower in fasted mice compared to non-fasted controls in WT and KI groups. However, no difference was observed between WT and KI groups in either fasted or non-fasted condition (**Fig 3A**). Significant muscle atrophy (~9% reduction compared to fed controls) was observed in gastrocnemius muscles of fasted mice, but the degree of muscle atrophy was similar between the WT and KI groups (**Fig 3B**). As expected, Chac1 KI increased glutathione level 23% compared to WT mice in the fed condition. Fasting reduced glutathione level 28% in gastrocnemius muscles of WT mice compared to non-fasted mice. Interestingly, depletion of glutathione by fasting was fully prevented by Chac1 inactivation in the KI mice (**Fig 3C**). These data indicate that fasting-induced glutathione depletion is likely mediated by CHAC1. However, preventing glutathione degradation by CHAC1 inactivation does not revert muscle atrophy by fasting, suggesting that preservation of glutathione alone is not sufficient to alleviate fasting-induced muscle atrophy.

### CHAC1 inactivation preserves glutathione but does not protect against Pan02 cancer induced muscle wasting

**Fig 4:**
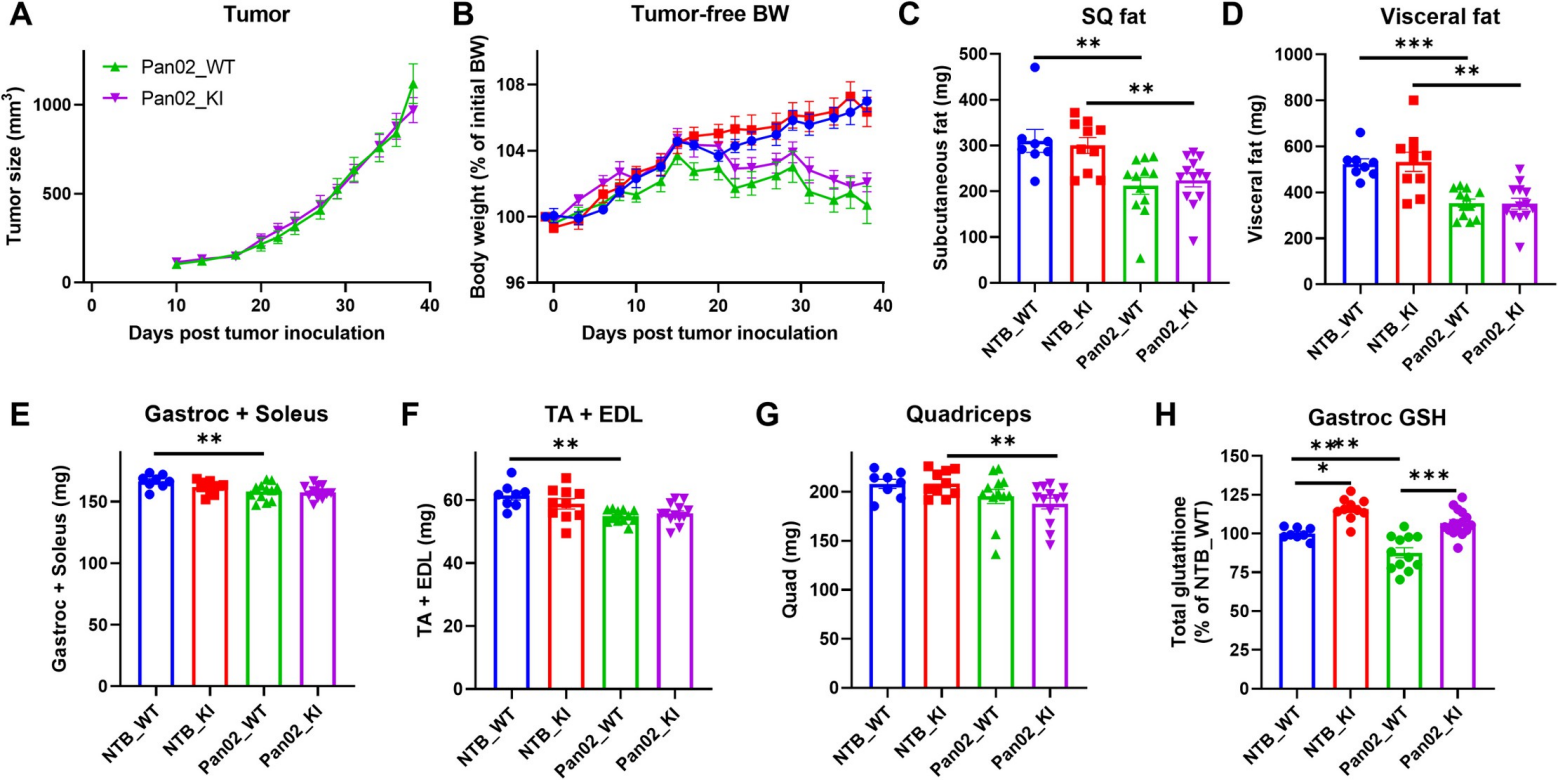
Chac1 inactivation preserves muscle glutathione but does not protect against muscle wasting in the Pan02 cachexia model. **A.** Tumor growth curves in Pan02 tumor-bearing WT and KI mice. **B.** Curves of estimated tumor-free body weights normalized by initial body weights on day 0. Weights of **C.** Subcutaneous (SQ) adipose tissue, **D.** Visceral adipose tissue, **E.** Gastrocnemius and soleus muscle, **F.** tibialis anterior (TA), and extensor digitorum longus (EDL) muscle, and **G.** Quadriceps muscle measured at takedown. **H.** Total glutathione measured in gastrocnemius muscles. Results are shown as mean ± SEM with individual data plotted. Statistical significances were indicated as: ^*^ *P* < 0.05, ^**^ *P* < 0.01, ^***^ *P* < 0.001.

To test the role of CHAC1 and glutathione in pathological muscle wasting condition, pancreatic adenocarcinoma Pan02 cells were subcutaneously implanted in Chac1 WT and KI mice to induce cachexia [30]. Tumor growth curve and terminal tumor weight were similar between the WT and KI groups (**Figs 4A and S4A**), indicating CHAC1 inactivation in the host does not impact tumor growth. Body weight started to separate between Pan02 tumor-bearing mice and NTB mice from day 20 after tumor implantation in both WT and KI groups (**Fig 4B**). At the end of study, significantly lower tumor-free body weight was found in Pan02 tumor-bearing mice (**S4B Fig**). No significant difference in tumor-free body weight was observed between WT and KI groups (**Figs 4B and S4B**). There was no significant difference in daily food intake between any groups (**S4C Fig**), suggesting food intake is not impacted by either Pan02 tumor or by CHAC1 deficiency in the host. Finally, subcutaneous and visceral adipose tissues and hindlimb skeletal muscle tissues, including gastrocnemius, soleus, TA, extensor digitorum longus (EDL), and quadriceps, were collected and weighed. Consistent with the body weight measurements, Pan02 tumor-bearing mice showed cachexia phenotype as evidenced by reduced adipose tissue mass and skeletal muscle mass in both WT and KI groups, but there was no significant difference between WT and KI mice (**Fig 4C–4G**). Despite lack of protection against either fat or muscle mass loss, Chac1 KI did increase glutathione level in gastrocnemius muscles ~16% in NTB KI mice as expected. Pan02 tumors reduced glutathione level ~12% in WT mice, which was brought up to 107% by Chac1 KI (**Fig 4H**). In line with the increased glutathione level in the KI mice, total and reduced form of antioxidant NADPH levels were also significantly increased in the NTB KI and Pan02 tumor bearing KI mice (**S5A-S5C Fig**), suggesting lower oxidative stress level in the KI mice. But unlike the case in the fasting study, glutathione level in Pan02 KI mice was not rescued to the same level as NTB KI mice, suggesting glutathione degradation mechanisms other than CHAC1 also contributed to Pan02 cancer induced glutathione depletion in skeletal muscle.

To explore the molecular mechanism underlying the observed phenotype, we analyzed expression of genes involved in muscle wasting pathways, including protein degradation, autophagy, oxidative stress, and ER stress. As expected, Pan02 tumor upregulated expression of several atrogenes including Fbxo32, Foxo1, Foxo3 in muscles, while there was no difference between WT and KI mice (**S6A Fig**). Expression of Nox4, a gene involved in production of superoxide, is increased by Pan02 tumor and suppressed by Chac1 KI, while expression of an antioxidant gene Sod1 is decreased by Pan02 tumor and increased by Chac1 KI (**S6B Fig**). These data consistently suggest that Chac1 KI suppressed Pan02 tumor induced oxidative stress but did not impact cachexia pathway. Interesting, we found expression of genes in glutathione synthesis pathway, including Gclc and Gclm, and glutathione oxidation gene Gpx4 is significantly suppressed by Chac1 KI (**S6C Fig**), suggesting a feedback inhibition of glutathione metabolism is induced by CHAC1 inactivation. Moreover, expression of autophagy genes Atg5 and Becn1, and gene in ER stress pathway Atf4 is upregulated by Chac1 KI (**S6D and S6E Fig**). The induction of autophagy and ER stress could be at least partially responsible for the lack of protection against muscle wasting by CHAC1 inactivation.

### CHAC1 inactivation preserves glutathione but does not protect against cisplatin induced muscle wasting

**Fig 5:**
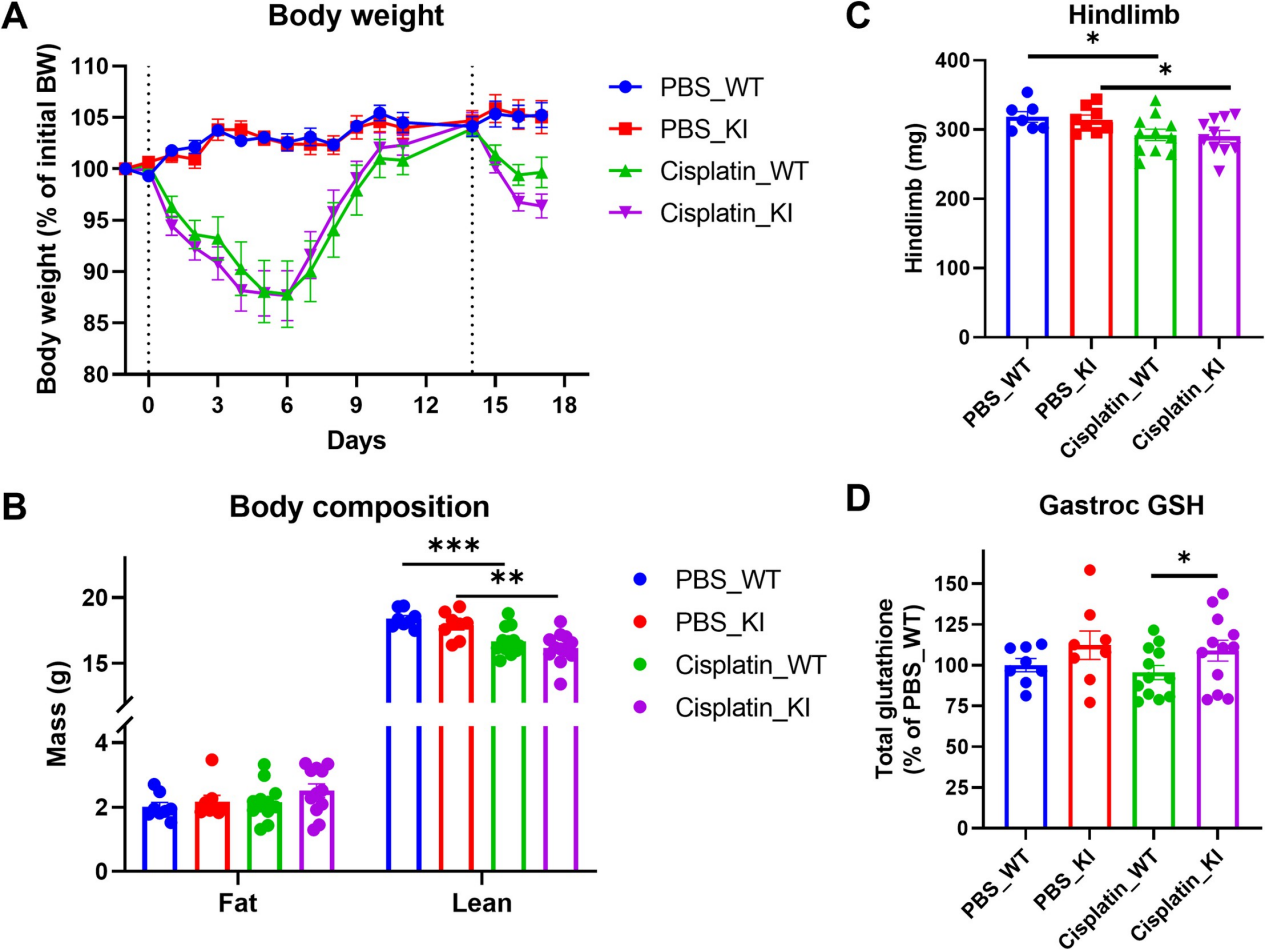
Chac1 inactivation preserves glutathione but does not protect against cisplatin induced muscle wasting. **A.** Curves of body weights normalized by initial body weights on day -1. **B.** Body compositions measured at the end of study. **C.** Weights of total hindlimb muscles, including gastrocnemius, TA, and quadriceps, measured at takedown. **D.** Total glutathione measured in gastrocnemius muscles. Results are shown as mean ± SEM with individual data plotted. Statistical significances were indicated as: ^*^ *P* < 0.05, ^**^ *P* < 0.01, ^***^ *P* < 0.001.

Chemotherapeutic agents, such as cisplatin, have been known to promote the progression of cachexia in cancer patients [33]. Cancer induced cachexia and chemotherapy induced cachexia may have distinct metabolic alterations [34]. Therefore, we tested the effects of CHAC1 inactivation in cisplatin induced cachexia. One dosing of cisplatin induced acute and drastic body weight loss in both WT and KI mice. The mice were allowed to fully recover in body weight after the initial dose before a second dose was given. No difference was observed between KI and WT mice in body weight loss either after the dosing or in the recovery phase (**Fig 5A**). Body compositional analysis by EchoMRI showed significant lean mass loss in cisplatin treated groups at the end of the study (**Fig 5B**). Consistently, total hindlimb muscle mass was also reduced in the treatment groups (**Fig 5C**), suggesting muscle wasting was induced in this model. Again, no significant difference was observed in lean or muscle mass between WT and KI mice, despite thatChac1 inactivation prevented cisplatin induced glutathione depletion in KI mice (**Fig 5D**).

Overall, these data support the notion that muscle wasting induced by the experimental conditions in this study may have multi-faceted underlying mechanisms and that preserving glutathione in skeletal muscle by CHAC1 inactivation alone has no protection against muscle wasting in chemotherapy induced cachexia model, consistent with the conclusion drawn in Pan02 cancer induced cachexia study.

## Discussion

By studying CHAC1 enzymatically inactive knock-in mice, we found inactivation of CHAC1 preserves glutathione in skeletal muscle under both physiological and pathological conditions. Although our results suggest that glutathione preservation may not have sufficient power to prevent muscle wasting induced by fasting, tumor, or chemotherapy, it is possible that preserving glutathione may have more beneficial impact on muscle function. A recent paper reported increased oxidative stress impaired muscle contractile function but increased muscle mass [35]. In a mouse model of oxidative stress (Sod1 KO mice), oxidative stress most significantly exacerbated cancer cachexia-induced muscle functional impairment, but not muscle mass loss, by disrupting neuromuscular junction [12]. Unfortunately, neither Pan02 cancer nor cisplatin induced cachexia model was found to manifest significantly impaired muscle function (data not shown) and thus not suitable to test the effect of CHAC1 inactivation on muscle function. Further investigations will be needed to test CHAC1 inactivation in a suitable cachexia model to assess the effect on muscle function.

Although CHAC1 inactivation preserves glutathione in both physiological and pathological conditions, other mechanisms contributing to glutathione degradation also need to be considered. Oxidized glutathione GSSG or glutathione conjugates that are secreted extracellularly can be degraded by GGT. Intracellular glutathione can also be degraded by CHAC2, another member of the CHAC family [24]. The observation that glutathione in Pan02 tumor-bearing Chac1 KI mice was not rescued to the same level as in NTB KI mice suggests additional mechanisms contributed to glutathione depletion in this model. Blocking all the glutathione degradation pathways might be needed to boost glutathione to sufficient level. It is also possible that a super pathophysiological level of glutathione is required to show beneficial effects, in which case blocking endogenous glutathione degradation may need to be combined with other strategies, such as nutritional supplementation or appetite stimulant, to achieve such high level of glutathione.

Our results do not invalidate the role of oxidative stress in muscle wasting as total glutathione level may not be directly correlated with oxidative stress level for the following reasons. First, glutathione exists in both reduced and oxidized forms where only the reduced form has antioxidant function. Under physiological conditions, reduced form (GSH) is 10–100 times more abundant than its oxidized form (GSSG) [23]. However, the ratio of GSH/GSSG may change dramatically under pathological conditions. As a result, increasing total glutathione in diseased condition may not necessarily increase its antioxidant capacity. Second, glutathione may have other functions beyond serving as an antioxidant, for example, as a reservoir for amino acids, like cysteine. This might be the case in fasting induced glutathione depletion, where free amino acids are deficient, that glutathione is degraded to replenish free amino acid level. Third, other endogenous antioxidants, such as NADPH, or antioxidant enzymes also contribute to oxidative stress. Direct measurements of oxidative stress parameters such as ROS/RNS level, protein carbonylation, and lipid peroxidation will be needed to find out the oxidative stress level in these models. It might also be worth further exploring and testing other targets in oxidative stress pathways for treating muscle wasting in cancer cachexia.

We found general upregulation of CHAC1 in various muscle wasting conditions. Besides being a glutathione degradation enzyme, CHAC1 may have other functions. Literature has reported CHAC1 as a proapoptotic gene involved in the unfolded protein response regulated by the ATF4-ATF3-CHOP pathway [25, 26, 36]. ER stress inducing chemicals and oxidized lipids significantly induce the expression of CHAC1 [25, 36]. Inhibition of cysteine transporter x_c_^-^ or deprivation of cysteine induces ER stress, ferroptosis and upregulation of CHAC1 [37, 38]. Additionally, CHAC1 is nutritionally regulated in response to refeeding [39] or high fat diet [40], in line with our data in the fasting study. These studies suggest CHAC1 may be transcriptionally induced in response to multiple mechanisms including oxidative stress, unfolded protein stress, or nutrition state. It is not known whether CHAC1 solely serves as a stress marker gene or plays a critical role in mediating these responses. In fact, our gene expression analysis suggested autophagy and ER stress pathway are activated in Chac1 KI muscle, suggesting CHAC1 may be involved in other pathways besides functioning as a glutathione degradation enzyme. Elucidating all the functions of CHAC1 and whether these functions are enzymatic activity dependent require further studies.

## Materials and methods

### Generation of *Chac1*^E116A^ knock-in mouse line

All animal studies were conducted under protocols approved by the Institutional Animal Care and Use Committee (IACUC). All animals were euthanized using CO_2_ inhalation followed by cervical dislocation when study endpoints or humane endpoints were met. Efforts were made to alleviate suffering. *Chac1*^E116A^ knock-in mouse line was generated on C57Bl/6J background by introducing the E116A (GAA>GCC) mutation into the exon 3 of Chac1 gene with CRSPR/Cas9. Two gRNAs were designed with the following sequences: gRNA1: AATGTGAGGGAAGCCGTGCT; and gRNA2: GT-GAGGGAAGCCGTGCTTGG. Genomic mutation was confirmed by PCR and sequencing using following primers: F1: TAGGTGTGGAATGTGTCTAGG; F2: CTAGAATGAACACAGGACC- AGC; R1: AGCTGCATGAAGTCTGCC; R2: CCCACAGAGCTGCATGAAG. Mouse zygotes were collected from superovulated donors, electroporated with Cas9/gRNA and a single strand DNA donor containing the E116A mutation and then transferred to surrogate females to generate founder animals. Selected founders were crossed with wild type animals for germline transmission. Three lines, including line 39 (E116A knock-in), line 24 (E116A knock-in), and line 40A (1 bp insertion), were selected for characterization. In the characterization study, body weights of homozygotes (HO), heterozygotes (HE) and littermate wild types (WT) were monitored for six weeks after mice were genotyped and weaned. Body composition was measured by EchoMRI machine (EchoMRI LLC) prior to takedown at 12~16 weeks old. Gastrocnemius, tibialis anterior (TA) muscles from left legs, heart, liver, and lung were isolated and weighed. Gastrocnemius muscle tissues were snap frozen in liquid nitrogen. Eventually, line 39 with E116A knock-in was selected for breeding and colony expansion. All the in vivo studies were conducted using this line.

### Fasting study in Chac1 KI mice

Female Chac1 WT and E116A knock-in mice at 12~13 weeks old were singly housed on chow diet at standard room temperature. The mice were randomized based on initial body weights and assigned into four groups, Fed_WT, Fed_KI, Fasted_WT, and Fasted_KI with 4–17 mice per group. The mice were deprived of food for 24 h before euthanasia. Gastrocnemius muscles were isolated, weighed, and snap frozen in liquid nitrogen.

### Pan02 tumor study in Chac1 KI mice

Littermate male Chac1 WT and E116A knock-in mice at 11~12 weeks old were singly housed on chow diet at standard room temperature. The mice were randomized based on initial body weights and assigned into four groups, NTB_WT, NTB_KI, Pan02_WT, and Pan02_KI with 8–15 mice per group. Murine pancreatic cancer Pan02 cells were cultured in high RPMI 1640 medium with 10% heat inactivated FBS and 1% pen/strep. Cells were resuspended in 1x PBS at density of 5×10^7^ cells/ml. Tumors were implanted in the mice by s.c. injection of 200 μl cell suspension with 25-gauge needles. Sham group received the same volume of sterile PBS. Mice were anesthetized under isoflurane inhalation during tumor implantation. Body and food weights were measured three times a week. Tumor measurement started at day 10 after tumor implantation using a digital caliper (Fisher Scientific, #06-664-16). Tumor volume was calculated as ½×L×W^2^, where L is the tumor length, and W is the tumor width. Based on the calculated volume, a density of 1.0 g/cm^3^ was used to estimate tumor weight. The mice were euthanized on day 38 after tumor implantation. Skeletal muscles, including gastrocnemius, soleus, TA, EDL, and quadriceps muscle, subcutaneous and visceral adipose tissue, heart, liver, lung, and tumors were collected and weighed at the end of the study. Gastrocnemius muscle tissues were snap frozen in liquid nitrogen.

### Cisplatin study in Chac1 KI mice

7–8 weeks old littermate female Chac1 WT and E116A knock-in mice were singly housed on chow diet at standard room temperature. The mice were randomized based on initial body weights and assigned into four groups, Vehicle_WT, Vehicle_KI, Cisplatin_WT, and Cisplatin_KI with 8–12 mice per group. Cisplatin (Sigma-Aldrich # 232120) dissolved in sterile water at 1 mg/ml was administrated by i.p. injection with an initial dose of 10 mg/kg and secondary dose of 5 mg/kg. Body weight and food weight were measured daily. Body composition was measured by EchoMRI machine (EchoMRI LLC) on day -1, 8, and 15. On day 17, mice were euthanized, and the following tissues were weighed and collected: gastrocnemius, soleus, TA, EDL, and quadriceps muscle, heart, and liver. Gastrocnemius muscle tissues were snap frozen in liquid nitrogen and stored at -80°C until use.

### Cell culture and plasmid transfection

HEK-293T cells were purchased from ATCC (Cat #CRL-3216). The cells were cultured in high glucose DMEM medium with 10% heat inactivated FBS and 1% pen/strep. The following constructs were used for transfection: pCDNA3.1 vector, pCDNA3.1-hCHAC1, pCDNA3.1-hCHAC1(E115Q), pCDNA3.1-hCHAC1(E115A). Cells seeded in 6-well plates were transfected with 2 ug of plasmid each well using Lipofectamine 2000 (Invitrogen #11668027) with 6 replicates. Two days after transfection, half of the samples were collected for Western blot analysis of CHAC1 expression and the other half for glutathione assay.

### Glutathione assay

Glutathione measurement was performed using an enzymatic assay kit (Sigma #CS0260) per manufacture’s protocol. For glutathione extraction from skeletal muscle tissues, frozen tissue was pulverized with ~50 mg of samples collected and weighed in homogenizing tubes. 10 volumes of 5% 5-sulfosalicylic acid (SSA) solution were added to each vial. For glutathione extraction from cultured cells, ~1 million cells were collected and 50 ul 5% SSA was added to lyse the cells. After homogenization, samples were centrifuged at 10,000 g for 10 min and the supernatants were collected. Samples were diluted 1–5 times before glutathione assay as needed. Standard curve was generated using the provided glutathione standard. Samples were run in duplicates. Total glutathione was measured by this kit and normalized by tissue weight or total protein amount extracted from same number of cultured cells.

### NADP/NADPH assay

NADP/NAPDH quantification was performed per manufacturer’s protocol (Sigma-Aldrich, Cat# MAK-038). Mouse quadricep muscle tissues were lysed (MPbio, Lysing Matrix D) and purified using 50kDa then 10kDa MWCO spin columns. Purified samples were split into two groups, direct measurement of sample for NADP_(total)_, and incubation at 60°C for 30 minutes to decompose NADP for measurement of NADPH. Absorbance was read at 450nm after 1 hour incubation with NADPH developer at room temperature. Protein concentration was measured by BCA in RIPA at the same volume to mg tissue ratio (10ul:1mg) as the assay. NADPH/NADP ratio was calculated by $Ratio=\frac{NADPH}{NADP_{\left(total\right)}-NADPH}$.

### Western blot of CHAC1

Protein was extracted from collected HEK-293T cells using RIPA lysis buffer (Thermo Scientific #89900). Total protein concentration was determined by BCA assay. ~30 ug total protein per well as loaded onto 4–12% gradient gels and transferred to nitrocellulose membrane. Membrane was blocked with 1x Blocker BSA in TBST (Thermo Scientific #37520), incubated with mouse polyclonal antibody against CHAC1 (Invitrogen #PA5-42212) at 1:1000 dilution and goat-anti-mouse secondary antibody at 1:5000 dilution (Invitrogen # 62–6520).

### Gene expression analysis by RT-PCR

Approximately 30 mg of pulverized frozen muscle tissue was used for RNA extraction using TRIzol (Invitrogen #15596) and chloroform method. RNA was purified using the Qiagen RNeasy Mini Kit. RNA concentration was measured by LUNATIC instrument. RNA was converted to cDNAs using High-Capacity RNA-to-cDNA™ Kit (Invitrogen #4387406). RT-PCR was performed using PCR Master Mix (2X) (Thermo Scientific #K0171) and Taqman probes from Thermo Fisher (see **S1 Table** for probe information). Expression of mouse gene Ppia or Gapdh was used for normalization.

### Statistical analysis

Statistical analyses for endpoints taken at a single time point were done by one-way ANOVA with a post-hoc Tukey HSD test if significant or Welch’s two sample T test. For longitudinal body weight, food intake, and tumor size measurements we fit longitudinal mixed effects models with fixed effects for treatment group and time and a random effect for animal ID. The models utilized an AR(1) covariance structure and contrasts were taken at specific time points to compare groups. Statistical significance was determined with p value < 0.05 and indicated by * P < 0.05, ** P < 0.01, *** P < 0.001. All analyses were performed in R 4.0.5.

## Supporting information

S1 Fig Inactivation of the human CHAC1 enzyme activity by E115A or E115Q mutation. **A.** Total glutathione measured in HEK-293T cells overexpressing vector, WT hCHAC1, E115A CHAC1 mutant, or E115Q CHAC1 mutant. Results are shown as mean ± SEM with individual data plotted. **B.** Western blot of CHAC1 in the lysates of cells used in A.
(TIF)

S2 Fig Confirmation of E116A knock-in in the N1 heterozygotes by genome sequencing. Replacement of nucleotides AA with CC led to mutation of amino acid glutamate to alanine in one allele.
(TIF)

S3 Fig Total glutathione measured in heart and liver tissues of WT and HO *Chac1*^E116A^ knock-in mice. Results are shown as mean ± SEM with individual data plotted.
(TIF)

S4 Fig Additional data in Pan02 cancer cachexia study. **A.** Tumor weights measured at takedown. **B.** Tumor-free body weights calculated based on terminal body weight and tumor weights measured in A. **C.** Daily food intake curves. Results are shown as mean ± SEM with individual data plotted. Statistical significance was indicated as: ^**^ *P* < 0.01.
(TIF)

S5 Fig NADP(H) measurement in muscles from Chac1 KI mice Pan02 cancer cachexia study. **A.** Total NADP, **B.** NADPH, and **C.** NADPH/NADP ratio measured in mouse quadriceps muscle tissues normalized by protein amount. Results are shown as mean ± SEM with individual data plotted. Statistical significance was indicated as: ^*^ *P* < 0.05, ^**^ *P* < 0.01.
(TIF)

S6 Fig RT-qPCR analysis of gene expression in muscles from Chac1 KI mice Pan02 cancer cachexia study. Expression of genes involved in **A.** Atrogenes, **B.** Oxidative stress, **C.** Glutathione metabolism, **D.** Autophagy, and **E.** ER stress were analyzed in gastrocnemius muscle tissues. Results are shown as mean ± SEM with individual data plotted. Statistical significance was indicated as: ^*^ *P* < 0.05, ^**^ *P* < 0.01, ^***^ *P* < 0.001.
(TIF)

S1 Table TaqMan probes for gene expression analysis. (TIF)
