# Supplementary figures and images for: CHAC1 inactivation is effective to preserve muscle glutathione but is insufficient to protect against muscle wasting in cachexia
Source: PLoS One. 2023 Apr 4;18(4):e0283806. doi: 10.1371/journal.pone.0283806 (PMC10072464; doi:10.1371/journal.pone.0283806)

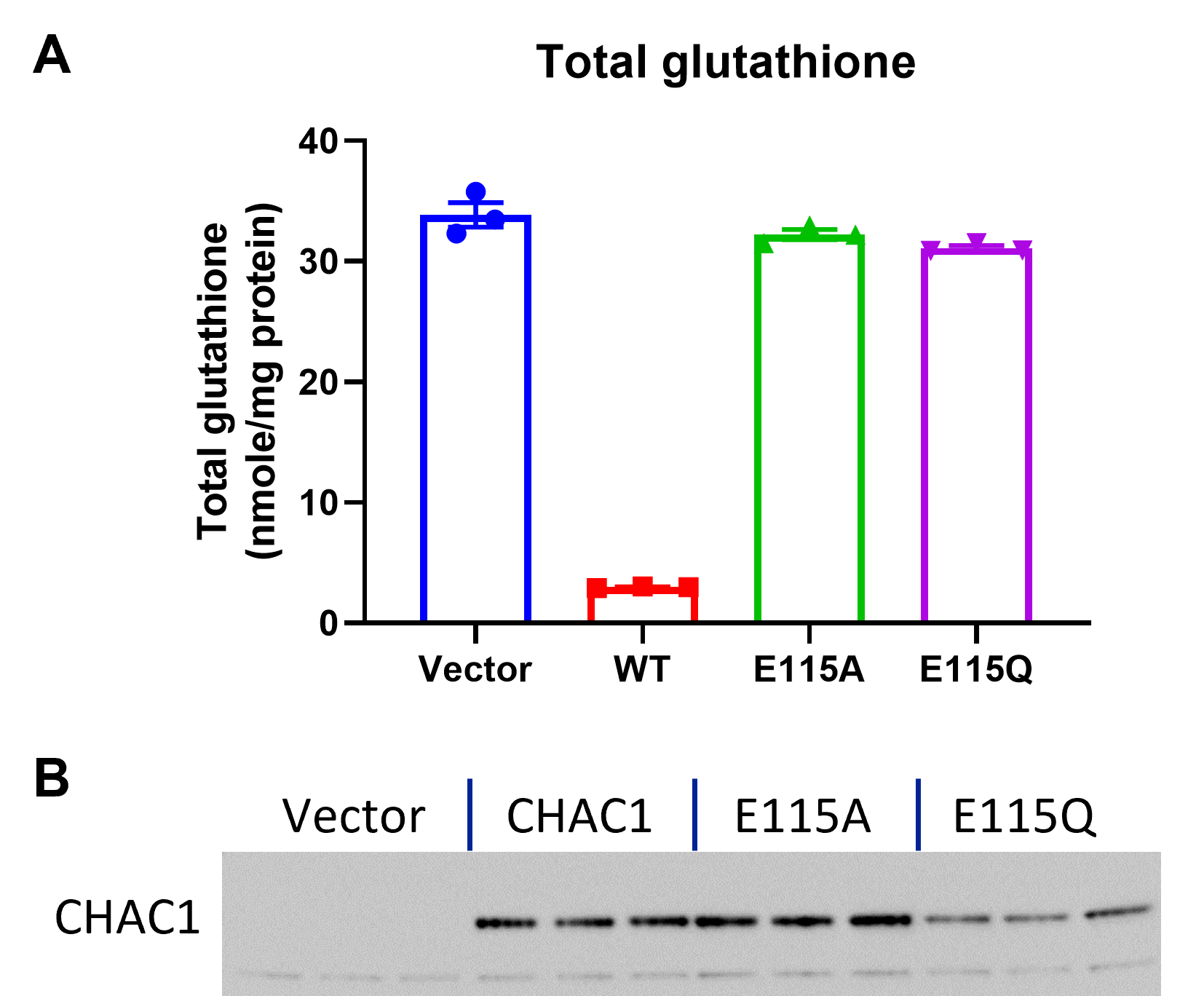

Supplement: S1 Fig — A. Total glutathione measured in HEK-293T cells overexpressing vector, WT hCHAC1, E115A CHAC1 mutant, or E115Q CHAC1 mutant. Results are shown as mean ± SEM with individual data plotted. B. Western blot of CHAC1 in the lysates of cells used in A. (TIF) [file pone.0283806.s001.tif]

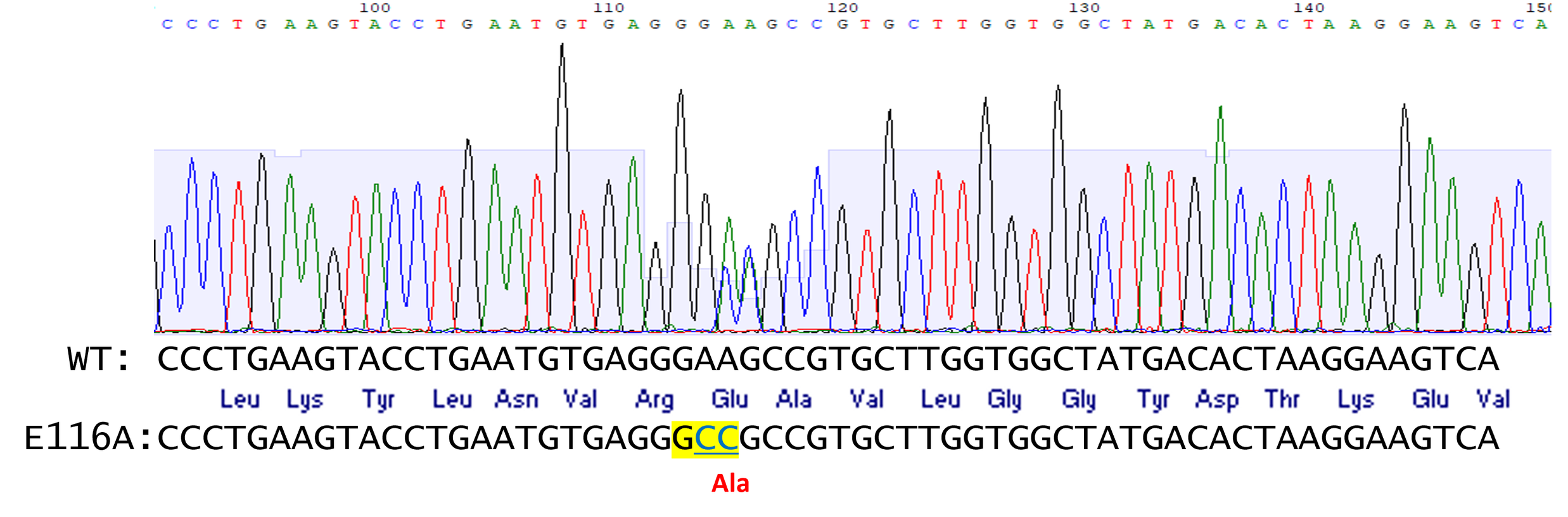

Supplement: S2 Fig — Replacement of nucleotides AA with CC led to mutation of amino acid glutamate to alanine in one allele. (TIF) [file pone.0283806.s002.tif]

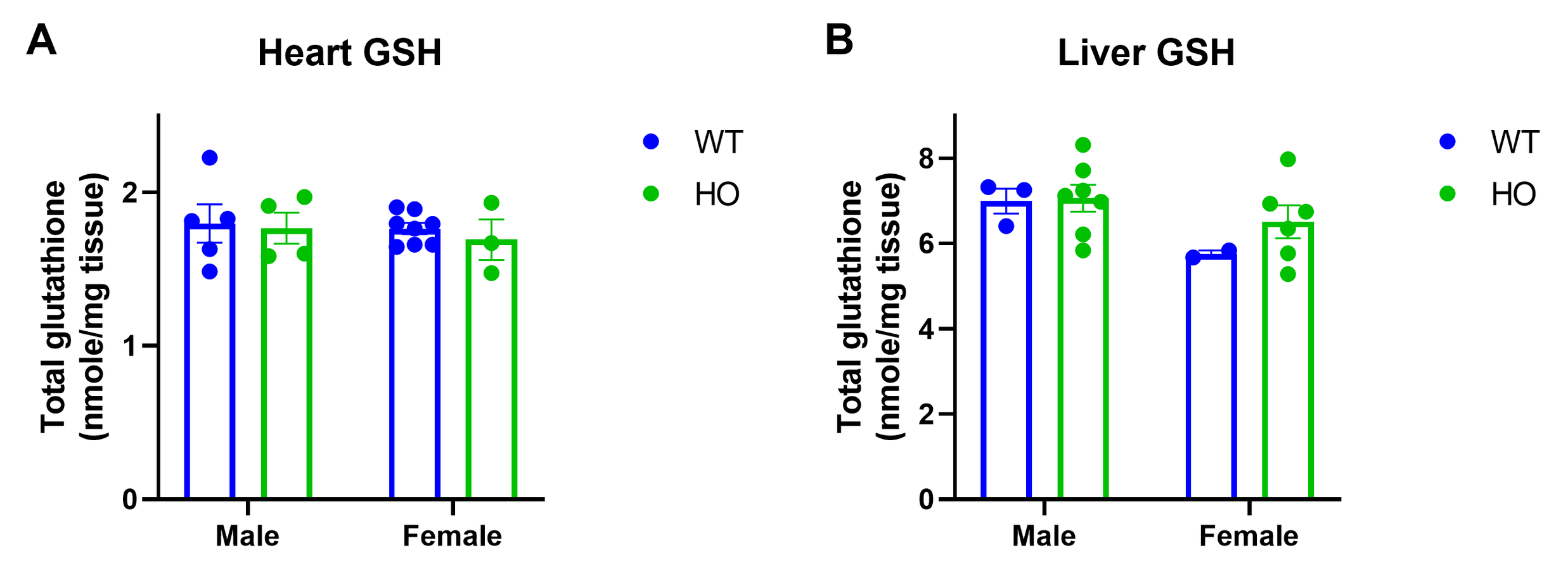

Supplement: S3 Fig — Results are shown as mean ± SEM with individual data plotted. (TIF) [file pone.0283806.s003.tif]

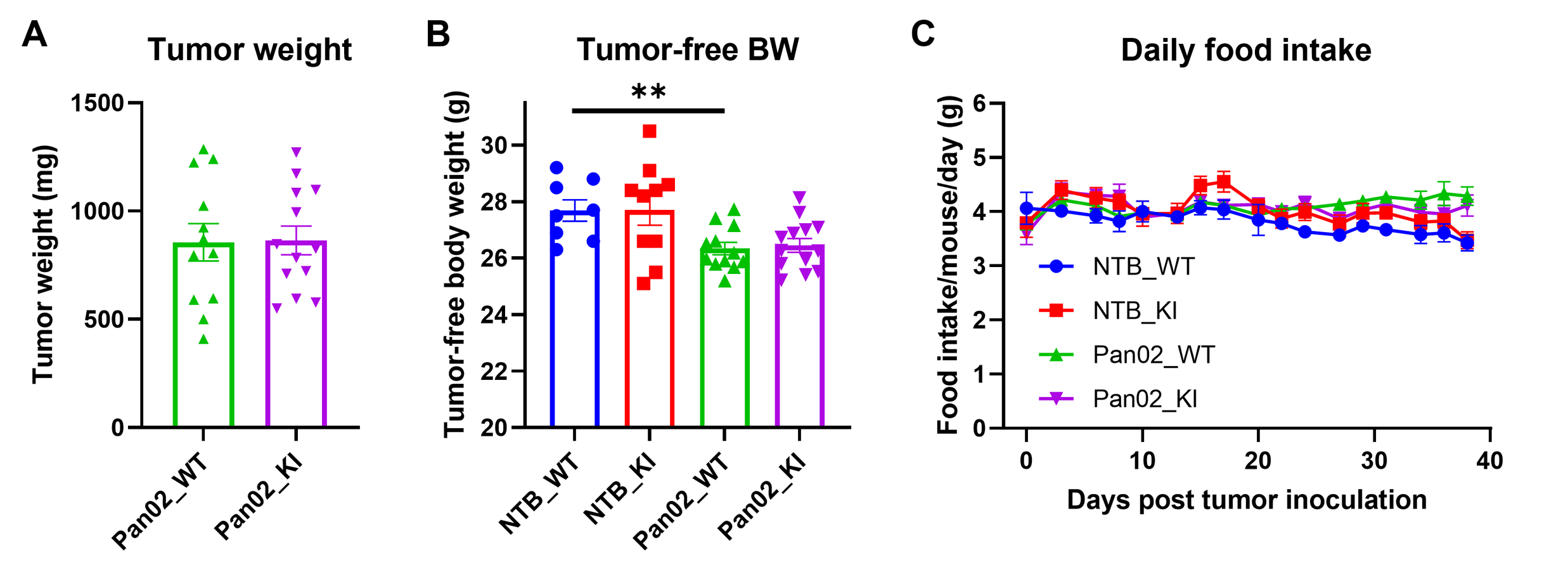

Supplement: S4 Fig — A. Tumor weights measured at takedown. B. Tumor-free body weights calculated based on terminal body weight and tumor weights measured in A. C. Daily food intake curves. Results are shown as mean ± SEM with individual data plotted. Statistical significance was indicated as: ** P < 0.01. (TIF) [file pone.0283806.s004.tif]

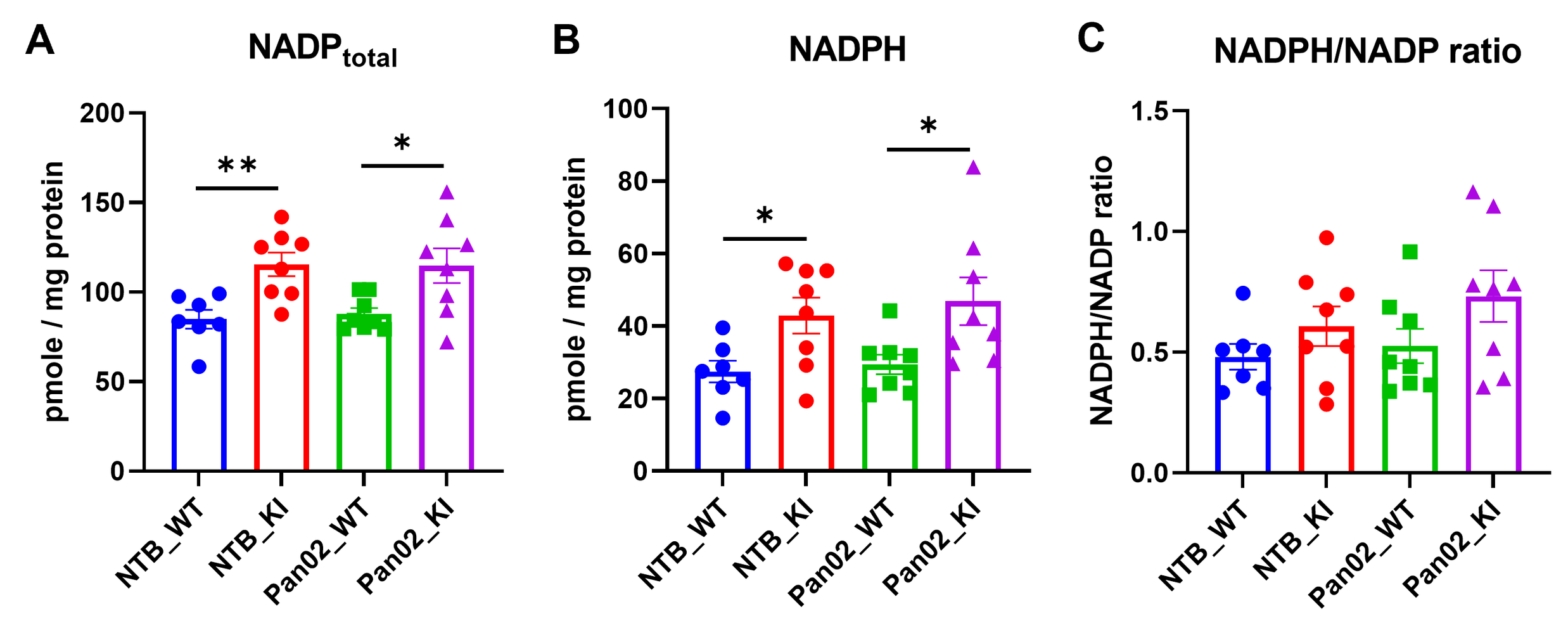

Supplement: S5 Fig — A. Total NADP, B. NADPH, and C. NADPH/NADP ratio measured in mouse quadriceps muscle tissues normalized by protein amount. Results are shown as mean ± SEM with individual data plotted. Statistical significance was indicated as: * P < 0.05, ** P < 0.01. (TIF) [file pone.0283806.s005.tif]

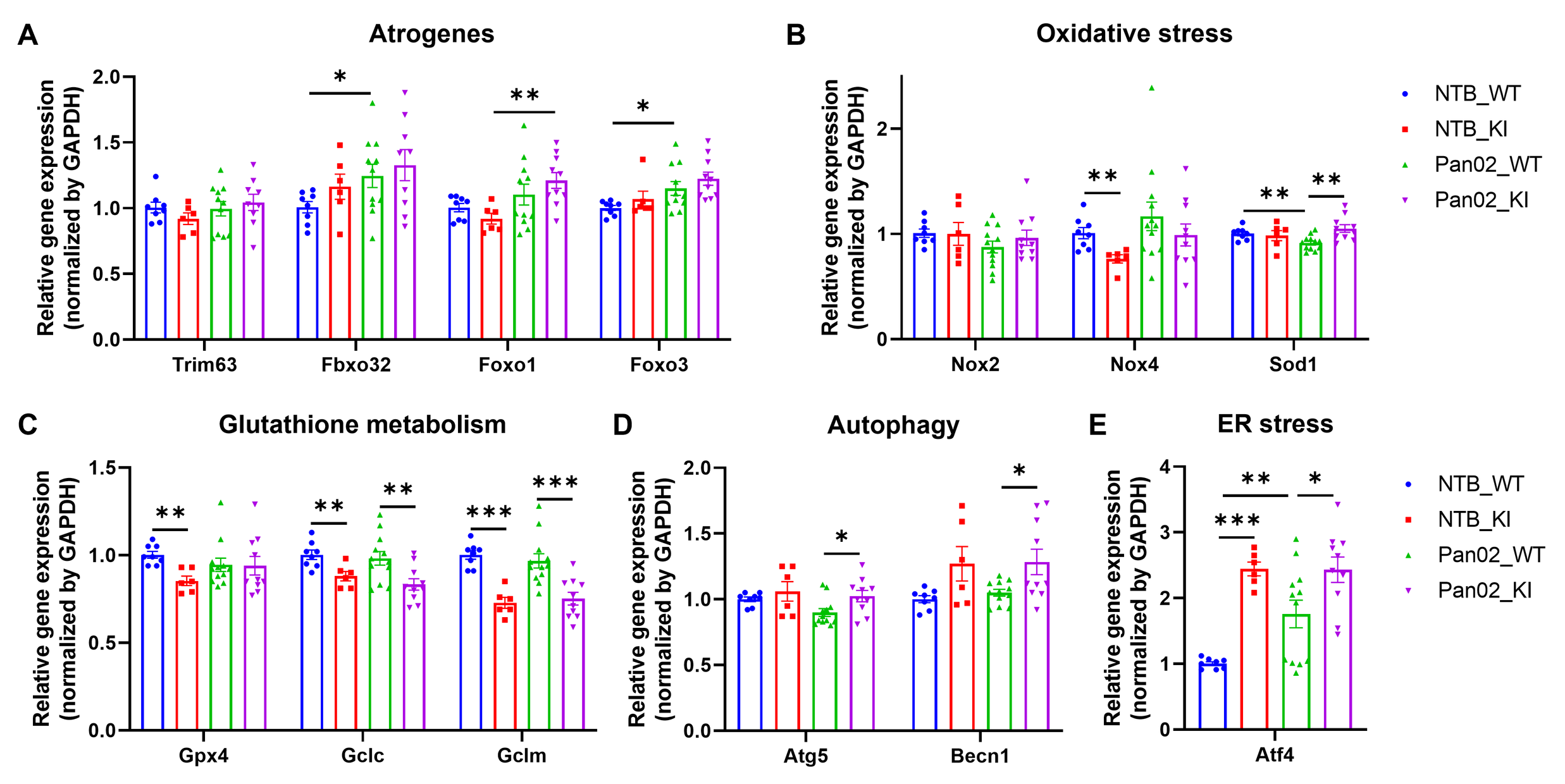

Supplement: S6 Fig — Expression of genes involved in A. Atrogenes, B. Oxidative stress, C. Glutathione metabolism, D. Autophagy, and E. ER stress were analyzed in gastrocnemius muscle tissues. Results are shown as mean ± SEM with individual data plotted. Statistical significance was indicated as: * P < 0.05, ** P < 0.01, *** P < 0.001. (TIF) [file pone.0283806.s006.tif]

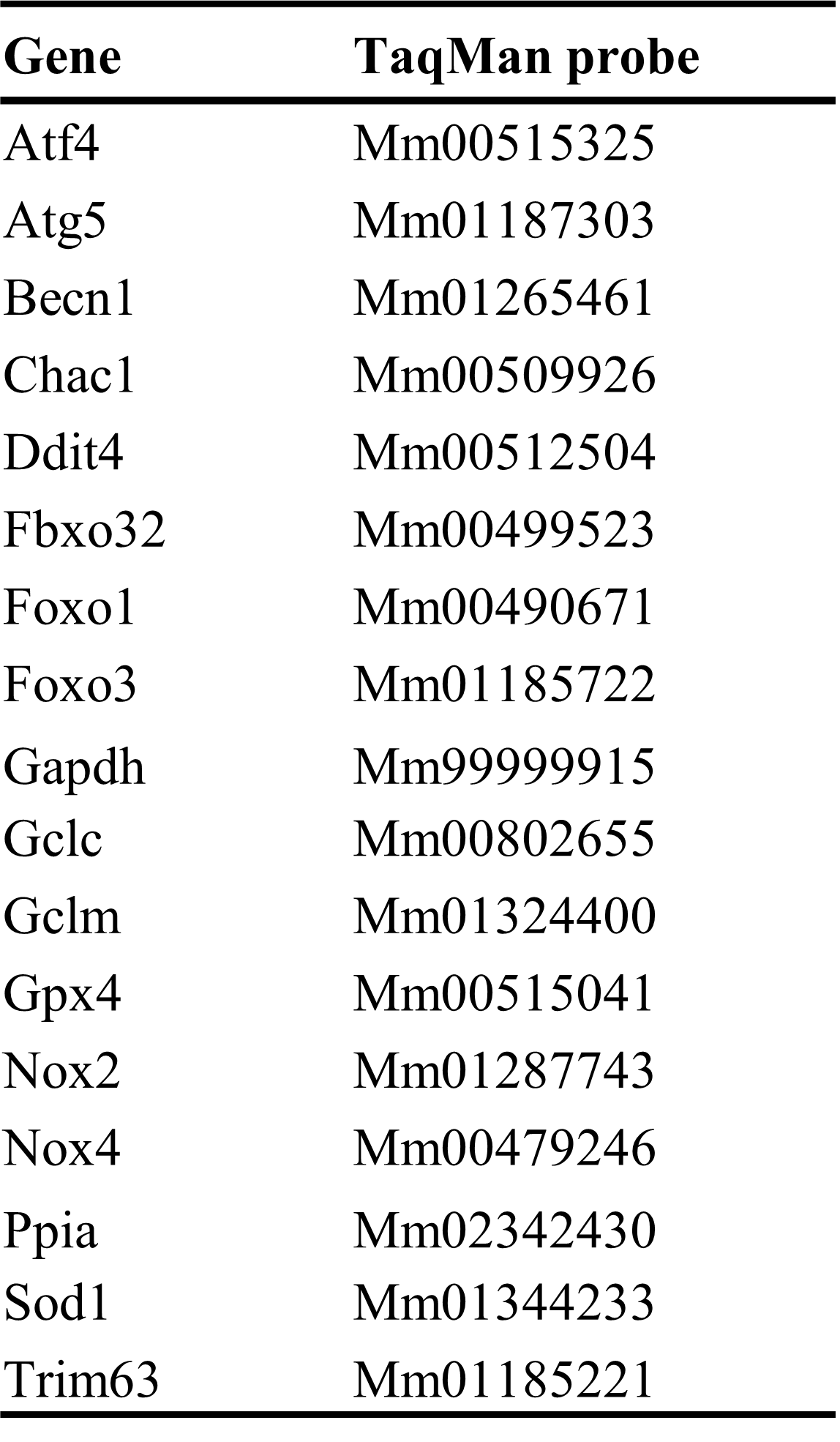

Supplement: S1 Table — (TIF) [file pone.0283806.s007.tif]
